# Deciphering the Elevated Lipid via CD36 in Mantle Cell Lymphoma with Bortezomib Resistance Using Synchrotron-Based Fourier Transform Infrared Spectroscopy of Single Cells

**DOI:** 10.3390/cancers11040576

**Published:** 2019-04-24

**Authors:** Sudjit Luanpitpong, Montira Janan, Kanjana Thumanu, Jirarat Poohadsuan, Napachai Rodboon, Phatchanat Klaihmon, Surapol Issaragrisil

**Affiliations:** 1 Siriraj Center of Excellence for Stem Cell Research, Faculty of Medicine Siriraj Hospital, Mahidol University, Bangkok 10700, Thailand; mj.montira@gmail.com (M.J.); jirarat_5696@hotmail.com (J.P.); npcrodboon@gmail.com (N.R.); p.klaihmon@gmail.com (P.K.); surapolsi@gmail.com (S.I.); 2 Synchrotron Light Research Institute (Public Organization), Nakhon Ratchasima 30000, Thailand; kthumanu@gmail.com; 3 Division of Hematology, Department of Medicine, Faculty of Medicine Siriraj Hospital, Mahidol University, Bangkok 10700, Thailand; 4 Bangkok Hematology Center, Wattanosoth Hospital, BDMS Center of Excellence for Cancer, Bangkok 10310, Thailand

**Keywords:** mantle cell lymphoma, bortezomib resistance, lipid metabolism, CD36, fourier transform infrared spectroscopy, single cells

## Abstract

Despite overall progress in improving cancer treatments, the complete response of mantle cell lymphoma (MCL) is still limited due to the inevitable development of drug resistance. More than half of patients did not attain response to bortezomib (BTZ), the approved treatment for relapsed or refractory MCL. Understanding how MCL cells acquire BTZ resistance at the molecular level may be a key to the long-term management of MCL patients and new therapeutic strategies. We established a series of de novo BTZ-resistant human MCL-derived cells with approximately 15- to 60-fold less sensitivity than those of parental cells. Using gene expression profiling, we discovered that putative cancer-related genes involved in drug resistance and cell survival tested were mostly downregulated, likely due to global DNA hypermethylation. Significant information on dysregulated lipid metabolism was obtained from synchrotron-based Fourier transform infrared (FTIR) spectroscopy of single cells. We demonstrated for the first time an upregulation of CD36 in highly BTZ-resistant cells in accordance with an increase in their lipid accumulation. Ectopic expression of CD36 causes an increase in lipid droplets and renders BTZ resistance to various human MCL cells. By contrast, inhibition of CD36 by neutralizing antibody strongly enhances BTZ sensitivity, particularly in CD36-overexpressing cells and de novo BTZ-resistant cells. Together, our findings highlight the potential application of CD36 inhibition for BTZ sensitization and suggest the use of FTIR spectroscopy as a promising technique in cancer research.

## 1. Introduction

Mantle cell lymphoma (MCL) is an aggressive non-Hodgkin lymphoma (NHL) that causes significant morbidity presumably due to inevitable development of drug resistance [1,2]. With the clinical pattern of poor response to conventional chemotherapy and frequent relapse, MCL remains the worst prognosis among all NHL subtypes [3,4]. Bortezomib (BTZ) is a Food and Drug Administration (FDA)-approved first-in-class proteasome inhibitor for the treatment of relapsed/refractory MCL. While a response rate of 30–50% in patients with relapsed disease could be achieved after BTZ treatment irrespective of their sensitivity to prior therapy, more than half of all MCL patients either did not initially respond to BTZ or subsequently acquired resistance [1,5]. Multiple combinations of BTZ and cytotoxic chemotherapy, immunomodulatory drug and/or histone deacetylase inhibitors have been intensively investigated in clinical trials to get higher response and better tolerability [6,7,8], implying that BTZ would remain one of the core drugs in treatment regimens and that understanding BTZ resistance is necessary for the long-term management of MCL patients and new therapeutic strategies.

Drug resistance is a manifestation of cancer, stemming from somatic mutations and genomic plasticity [9]. In this regard, numerous mechanisms associated with molecular changes of drug efflux pumps, drug metabolizing enzymes and apoptosis regulatory pathways have been shown to protect various cancers against chemotherapy [10,11,12]. Although the information on BTZ resistance in MCL is still very limited, certain characteristics of BTZ-resistant MCL have previously been reported, namely uncontrolled plasmacytic differentiation and activation of B-cell receptor (BCR) signaling [13,14]. Since drug resistance is a multi-factorial phenomenon, we established de novo BTZ-resistant MCL cells and performed large-scale expression analyses at the transcriptional and macromolecular levels using sets of quantitative polymerase chain reaction (PCR) and synchrotron-based Fourier transform infrared (FTIR) spectroscopy.

In the present study, we profiled putative cancer-related genes involved in drug resistance and cell survival and observed that most of the tested genes were unexpectedly downregulated in BTZ-resistant MCL. Further single cell analysis with FTIR spectroscopy, however, provided significant information on the dysregulation of lipid in highly resistant cells, leading us to the discovery of an alteration in CD36 receptor that modulates lipid homeostasis. Our findings could be important in understanding BTZ resistance and in developing novel effective adjuvant for MCL.

## 2. Results

### 2.1. Establishment of BTZ-Resistant MCL Cells and Their Sensitivity to BTZ

**Figure 1:**
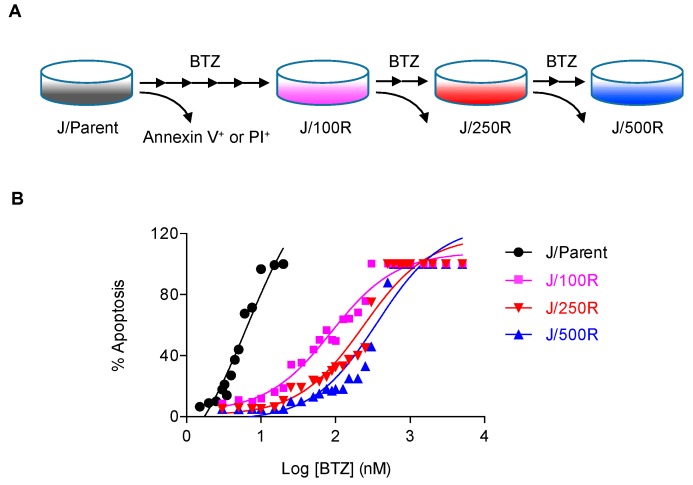
Resistant phenotype of de novo bortezomib (BTZ)-resistant mantle cell lymphoma (MCL) cells in comparison to parental MCL cells. (**A**) Schematic diagram for the establishment of BTZ-resistant MCL cells from parental (J/Parent) cells by stepwise selection method. (**B**) J/Parent cells and BTZ-resistant cells at different degrees (J/100R, J/250R and J/500R) were treated with various concentrations of BTZ (0–5000 nM) and apoptosis was determined by Hoechst 33342 at 24 h. Dose-response curves were generated on a logarithmic scale of drug concentration as opposed to a linear scale of percentage of apoptosis. Lethal concentration 50 (LC50) of BTZ in parental and resistant cells were then calculated from the plot and compared.

BTZ-resistant MCL cells with acquired resistance at different degrees were established from BTZ-sensitive human MCL-derived Jeko-1 (J/Parent) cells after sequential treatments with BTZ, i.e., up to 500 nM, and designated as J/BTZ100R, J/BTZ250R and J/BTZ500R (Figure 1A). The sensitivity of parental and resistant cells to BTZ at 24 h was first evaluated by Hoechst 33342 assay. Hoechst dye stains genomic DNA, allowing the determination of morphologic apoptosis of chromatin condensation and DNA fragmentation. Dose-response curve of BTZ-induced apoptosis was plotted (Figure 1B) and lethal concentration 50 (LC50), which causes half of the cells to undergo apoptosis, was calculated from the plot. The LC50 value (mean ± SE) for parental J/Parent cells was 6.39 ± 1.59 nM versus 87.81 ± 1.14, 236.30 ± 1.22 and 361.10 ± 1.28 nM for resistant J/BTZ100R, J/BTZ250R and J/BTZ500R, respectively. Resistance index (*R*) was then calculated according to the relation: *R* = LC50 resistant cells/LC50 parental cells. Therefore, the degrees of resistance in established resistant cells were approximately 15, 40 and 60-fold more resistant to BTZ than the parental cells.

### 2.2. Gene Expression Profiling of BTZ-Resistant MCL

**Figure 2:**
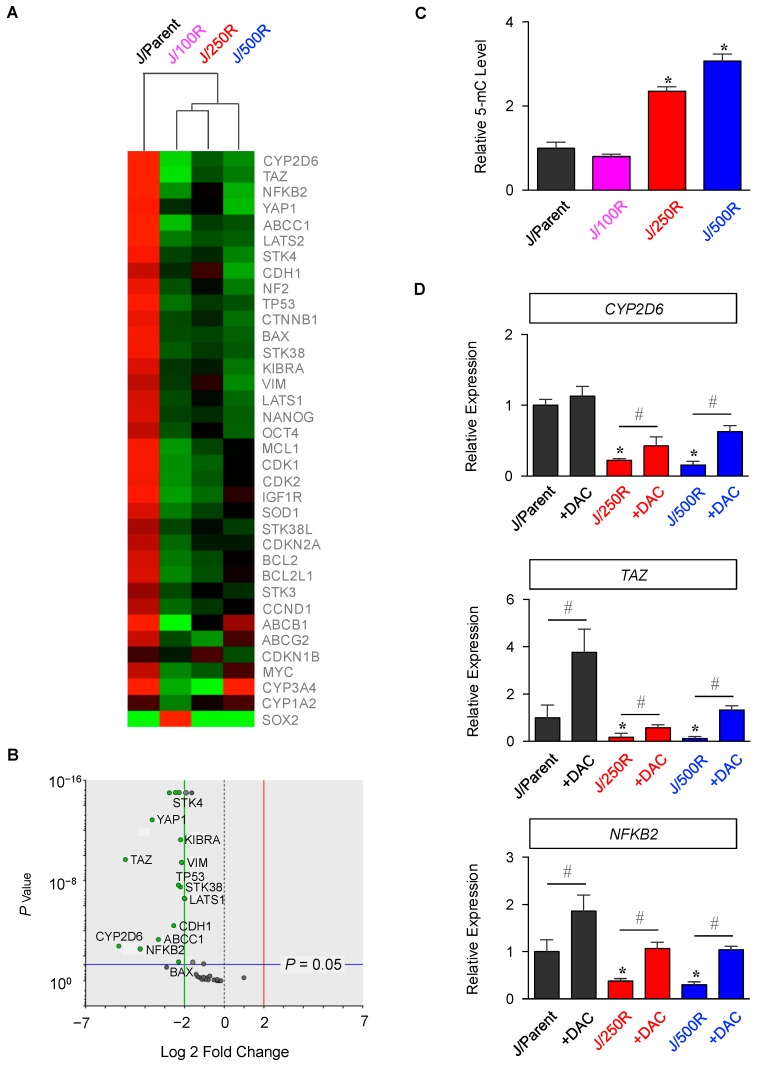
Analysis of mRNA expression of putative cancer-related genes involved in drug resistance and cell survival of MCL by quantitative real-time PCR. (**A**) Data from three independent experiments were normalized to house-keeping *GAPDH*, averaged and represented in heatmap in the three-color scale of red (upper), black (midpoint) and green (lower). (**B**) Volcano plot of differentially expressed genes comparing J/Parent and J/500R cells with fold change >2 and *p* < 0.05; two-sided Student’s *t*-test. (**C**) Global DNA methylation was evaluated by measurement of 5-methylcytosine (5-mC). Relative absorbance (OD450) of samples to parental cells are shown. Data are mean ± SD (*n* = 3). * *p* < 0.05 versus J/Parent cells; two-sided Student’s *t*-test. (**D**) J/Parent, J/250R and J/500R cells were sequentially treated with 5-aza-2′-deoxycytidine (DAC) (1 μM) for 72 h and mRNA expression of *CYP2D6*, *TAZ* and *NFKB2* were evaluated by quantitative real-time PCR. Data are mean ± SD (*n* = 3). * *p* < 0.05 versus J/Parent cells; two-sided Student’s *t*-test. ^#^ *p* < 0.05 versus non-treated cells; two-sided Student’s *t*-test.

In order to identify genes and pathways important in the development of BTZ resistance, we profiled putative cancer-related genes involving in drug resistance and cell survival of MCL using sets of quantitative PCR. These genes were grouped into: (i) Drug efflux pumps *ABCB1*, *ABCC1* and *ABCG2* [10]; (ii) drug metabolizing enzymes *CYP1A2*, *CYP2D6* and *CYP3A4* [11]; (iii) apoptosis regulators *BCL2*, *BCL2L1*, *MCL1*, *BAX* and *TP53* [12]; (iv) cell cycle regulators *CCND1*, *CDK2*, *CDK4*, *CDKN18* and *CDKN2A* [15]; (v) cell survival and growth factors *NFKB2*, *MYC*, *SOD1*, *STK38*, *STK38L*, and *IGF1R* [16,17,18]; (vi) Hippo pathway components *AMOT*, *NF2*, *LATS1*, *LATS2*, *STK3*, *STK4*, *YAP1*, *TAZ* and *KIBRA* [19]; (vii) epithelial–mesenchymal transition (EMT) regulators *SNAIL2*, *VIM*, *ZEB1*, *CTNBB1*, *CCND1* and *CDH1* [20]; and (viii) pluripotent genes *OCT4*, *NANOG* and *SOX2* [21]. Gene expression levels normalized to the housekeeping *GAPDH* comparing parental J/Parent cells and BTZ-resistant J/100R, J/250R and J/500R cells were averaged, clustered and represented in a heatmap. Figure 2A shows that most of the tested genes were unexpectedly downregulated in all resistant cells. Next, a volcano plot was created to better visualize the differential gene expression in J/Parent versus J/500R cells using more than two-fold change at *p* < 0.05 filter criteria. Figure 2B shows that 34% of these genes, including the well-known drug metabolizing enzyme *CYP2D6*, proto-oncogenes *TAZ* and *YAP1*, drug efflux pump *ABCC1* and survival transcription factor *NFKB2* were significantly downregulated in J/500R cells when compared to parental cells. One important mechanism for gene regulation is epigenetic alterations, particularly DNA methylation of CpG dinucleotides [22]. To investigate the plausible linkage between DNA methylation and the observed gene silencing in BTZ-resistant cells, we evaluated the global DNA methylation status by specifically measuring the level of 5-methylcytosine (5-mC), which is the most common epigenetic mark and an important transcriptional repressor [23]. An extensive increase in 5-mC was found in BTZ-resistant J/250R and J/500R cells when compared to parental cells in a dose-dependent manner (Figure 2C), indicating the hypermethylation status of J/250R and J/500R cells. Treatment of these BTZ-resistant cells with hypomethylating agent 5-aza-2′-deoxycytidine (DAC) significantly reactivated their expression of top-ranked differentially expressed genes *CYP2D6*, *TAZ* and *NFKB2* (Figure 2D), suggesting that hypermethylation might be in part responsible for such gene expression perturbation.

### 2.3. FTIR Signatures of BTZ-Resistant MCL

**Figure 3:**
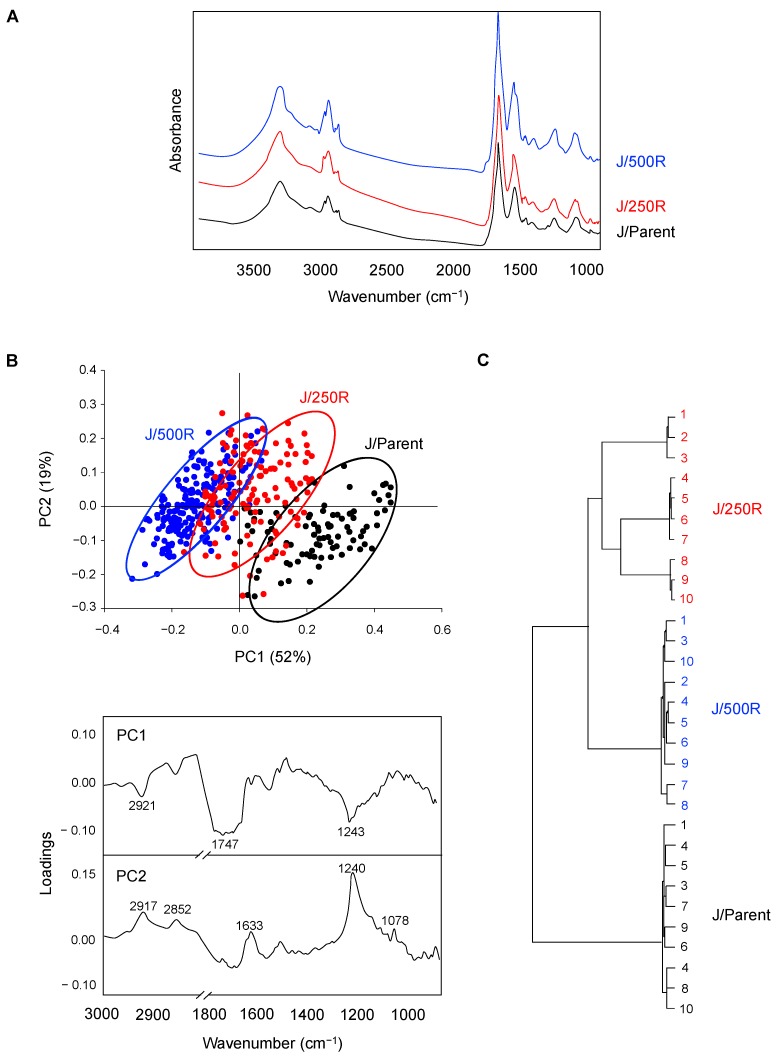
FTIR spectral signatures of parental and BTZ-resistant MCL cells. (**A**) Mean FTIR spectra of the parental J/Parent cells and BTZ-resistant J/250R and J/500R cells in the wavelength range of 4000 to 800 cm^−1^. (**B**) (top) Two-dimensional principal component analysis (PCA) score plot of all recorded FTIR spectra of J/Parent, J/250R and J/500R cells. Black dots represent J/Parent spectra, while black diamonds represent J/250R and grey triangles represent J/500R cells. Eclipses depicted in the plot define confidence limit, of which 95% of the data are allocated. (Bottom) Score loadings of PC1 (upper) and PC2 (lower) to identify the variables corresponding to wavelength number. (**C**) Unsupervised hierarchical classification of all recorded J/Parent, J/250R and J/500R spectra in the range of 3000–2800 cm^−1^ to 1750–800 cm^−1^.

FTIR spectroscopy is a sensitive technique capable of providing significant information on the biochemical content of cellular macromolecules, such as proteins, lipids, nucleic acids and carbohydrates, that could be linked to cellular organization and physiological state of the cells [24,25]. Figure 3A shows the mean FTIR spectra of the parental J/Parent cells and BTZ-resistant J/250R and J/500R cells recorded from more than 200 single cells in the mid-IR region of 4000 to 800 cm^−1^. Principle component analysis (PCA) is one of the most common multivariate techniques that concentrates the source of variability in the data into few PCs. The PCA of spectroscopic data provide two types of information: (i) visualization of clustering of similar spectra within datasets in scatter plots; and (ii) identification of variables (spectral bands representing various molecular groups within the samples) in loading plots. Figure 3B (top) shows the scatter plot of PC1 (PC score; 52%) against PC2 (19%) that could separate the parental cells from BTZ-resistant cells. In brief, J/Parent cells clustered in the positive direction of PC1, while BTZ-resistant cells, particularly J/500R cells, were mostly in the negative direction of PC1 in correlation with the spectral region centered at 2921 (C–H total lipid), 1747 (C=O ester lipid) and 1243 (nucleic acid) cm^−1^ (Figure 3B, bottom). For J/250R cells and J/500R cells, the separation along PC2 can also be explained by the positive loading for PC2 in the spectral region centered at 2917 and 2850 (C–H lipid), 1633 (β-sheet amide I), 1240 (nucleic acid) and 1078 (glycoprotein and carbohydrate region) cm^−1^. Hierarchical cluster analysis for the spectral regions from 3000–2800 cm^−1^ to 1750–800 cm^−1^ was performed as a second method to classify and determine the similarity of FTIR spectra of J/Parent, J/250R and J/500R cells. Figure 3C shows that the FTIR spectra of J/250 and J/500R cells harbored significant similarities (first group) whereas the J/Parent cells were clustered separately as a second group, indicating that FTIR spectral signatures could clearly distinguish BTZ-resistant cells from the parental cells.

**Figure 4:**
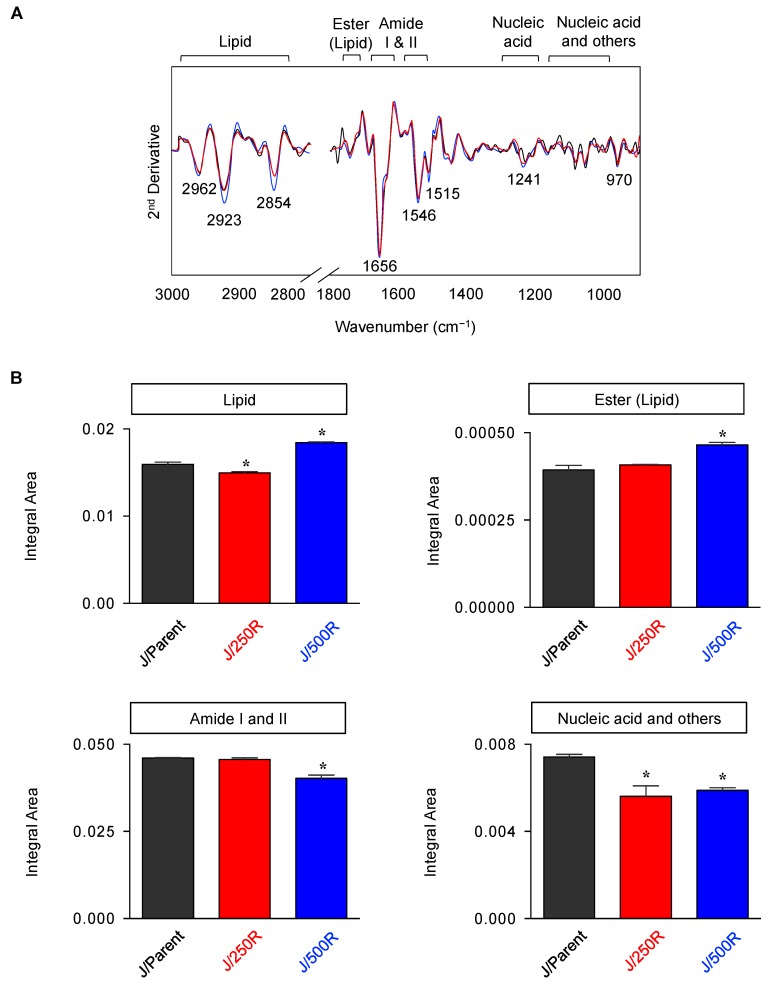
Spectral assignments and quantification of biochemical contents in parental and BTZ-resistant cells using integral area. (**A**) The second derivative spectra obtained from the mean FTIR spectra of the parental J/Parent cells and BTZ-resistant J/250R and J/500R cells in the wavelength range of 3000–2800 cm^−1^ to 1750–800 cm^−1^. Band assignments for the regions of lipid, ester (lipid), amide I, amide II, nucleic acid (DNA/RNA) and nucleic acid, glycoprotein and other carbohydrate (nucleic acid and others) were illustrated. (**B**) Integral area of total lipid, ester (lipid), combined amide I and II and nucleic acid and others, with observed changes, were plotted. Data are mean ± SD (*n* = 3). * *p* < 0.05 versus J/Parent cells; two-sided Student’s *t*-test.

To gain more insight into the differences between parental and BTZ-resistant cell lines, various biochemical contents of J/Parent, J/250R and J/500R cells were compared using integral areas of specified FTIR spectral regions corresponding to C–H from lipid (3000–2800 cm^−1^), C=O ester primarily from lipid (1750–1735 cm^−1^), amide I protein (1670–1600 cm^−1^), amide II protein (1560–1500 cm^−1^), P=O phosphodiester bond from nucleic acid (1260–1200 cm^−1^), C–C from nucleic acid and C–O from glycoprotein and other carbohydrate (nucleic acid and others; 1180–990 cm^−1^) and RNA (975–950 cm^−1^), as illustrated in Figure 4A (see also Supplementary Table S1). A remarkable increase of total lipid and ester (lipid) and decrease of amides and nucleic acid and others were observed in highly BTZ-resistant J/500R cells when compared to J/Parent cells (Figure 4B). RNA and solely nucleic acid contents were found to be unchanged. These discrepancies in biochemical contents between parental and BTZ-resistant cells might be due to the metabolic reprogramming that is an emerging hallmark of cancer and an attractive target in novel therapeutic strategies for cancer treatment.

### 2.4. Elevated Lipid Content and CD36 Upregulation in BTZ-Resistant MCL

**Figure 5:**
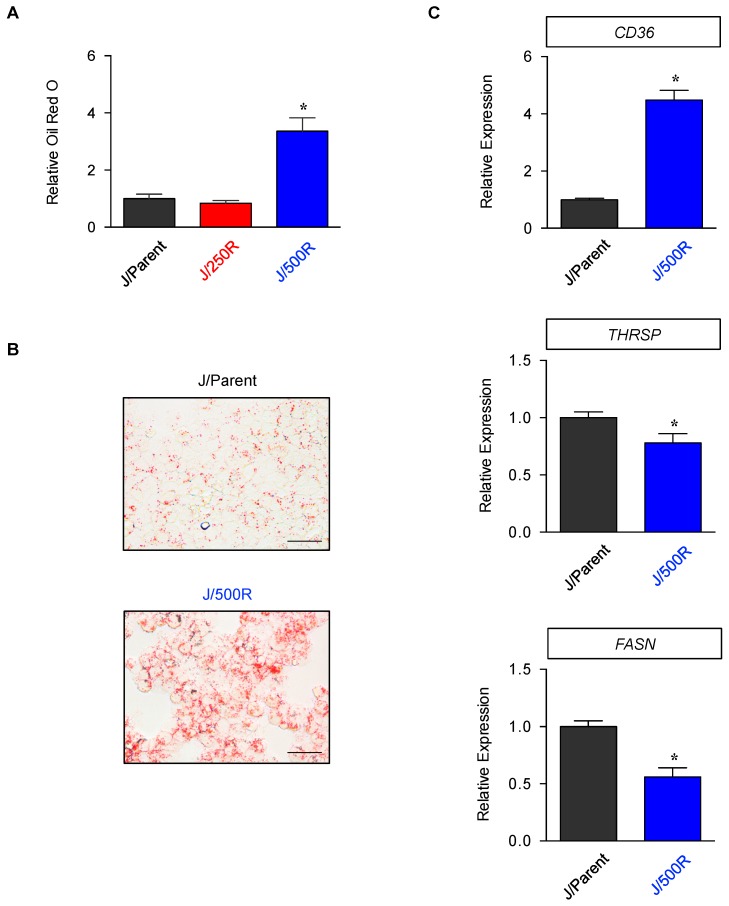
Determination of lipid droplets and expression of lipid markers in parental and BTZ-resistant cells. (**A**,**B**) Parental J/Parent cells and BTZ-resistant J/250 and J/500R cells were fixed and stained with oil red O. The stained cells were then visualized under a microscope or extracted with isopropanol for quantification. (**A**) Relative absorbance (OD510) of samples to parental cells are shown. Data are mean ± SD (*n* = 4). * *p* < 0.05 versus J/Parent cells; two-sided Student’s *t*-test. (**B**) Representative micrographs of oil red O-stained J/Parent and J/500R cells. Scale bar = 50 µm. (**C**) mRNA expression of fatty acid synthase (*FASN*), thyroid hormone responsive spot 14 (*THRSP*) and *CD36* using quantitative real-time PCR in J/Parent cells in comparison to J/500R cells. Data are mean ± SD (*n* = 4). * *p* < 0.05 versus J/Parent cells; two-sided Student’s *t*-test.

Lipids, i.e., long chain fatty acids and cholesterols, are essential components of biological membranes and are also used for energy storage, production and cellular signaling [26,27]. Enhanced uptake of exogenous lipids and/or overactivated endogenous synthesis provide rapidly proliferating tumor cells with energy and biomass component, conferring both growth and survival advantages that are associated with carcinogenesis and tumor progression [28,29]. Having demonstrated an increase in cellular lipid composition in BTZ-resistant J/500R cells from FTIR spectral analysis, we next examined the stored lipids using hydrophobic oil red O dye, which binds to lipid droplets due to their nonpolar nature. Figure 5A shows that J/500R cells had more than three-fold higher in oil red O staining when compared to parental cells. Representative micrographs of oil red O-stained cells show that an accumulation of lipid droplets (red colored dots) could be apparently visualized in J/500R cells (Figure 5B).

**Figure 6:**
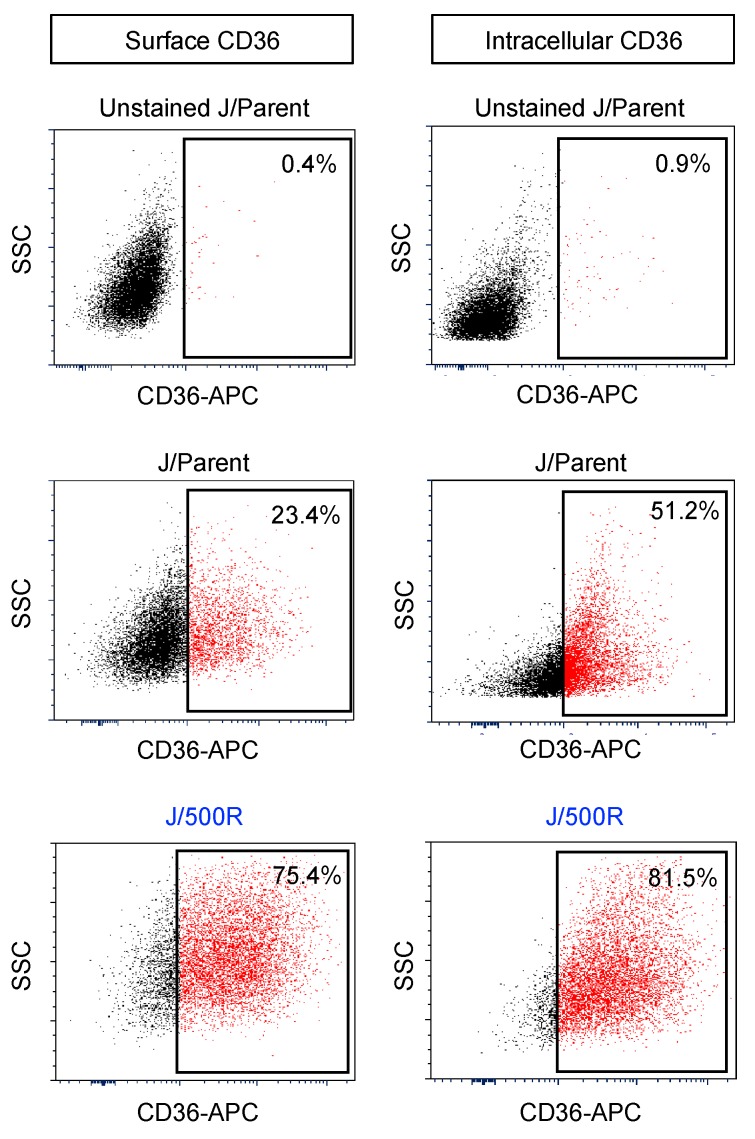
CD36 is overexpressed in BTZ-resistant cells. Flow cytometry analysis of surface (left) and intracellular (right) CD36 on parental J/Parent cells and BTZ-resistant J/500R cells. Percentage of CD36-positive cells (box) were determined based on their internal negative control (unstained cells).

We assessed the gene expression of markers of de novo fatty acid synthesis (*FASN*) and thyroid hormone responsive spot 14 (*THRSP*) and exogenous fatty acid uptake *CD36* [30,31] and observed a dramatic upregulation of *CD36* with subtle changes in *FASN* and *THRSP* in J/500R cells when compared to parental cells (Figure 5C). Hence, we postulated that an increase in lipids in BTZ-resistant J/500R cells was likely mediated by an increase in exogenous uptake via CD36 receptor. Flow cytometric analysis further revealed an increase in CD36 protein expression on both cell surface and intracellular compartment of J/500R cells when compared to parental cells (Figure 6).

### 2.5. CD36 Renders MCL Cells to BTZ Resistance and Is an Attractive Co-Target

**Figure 7:**
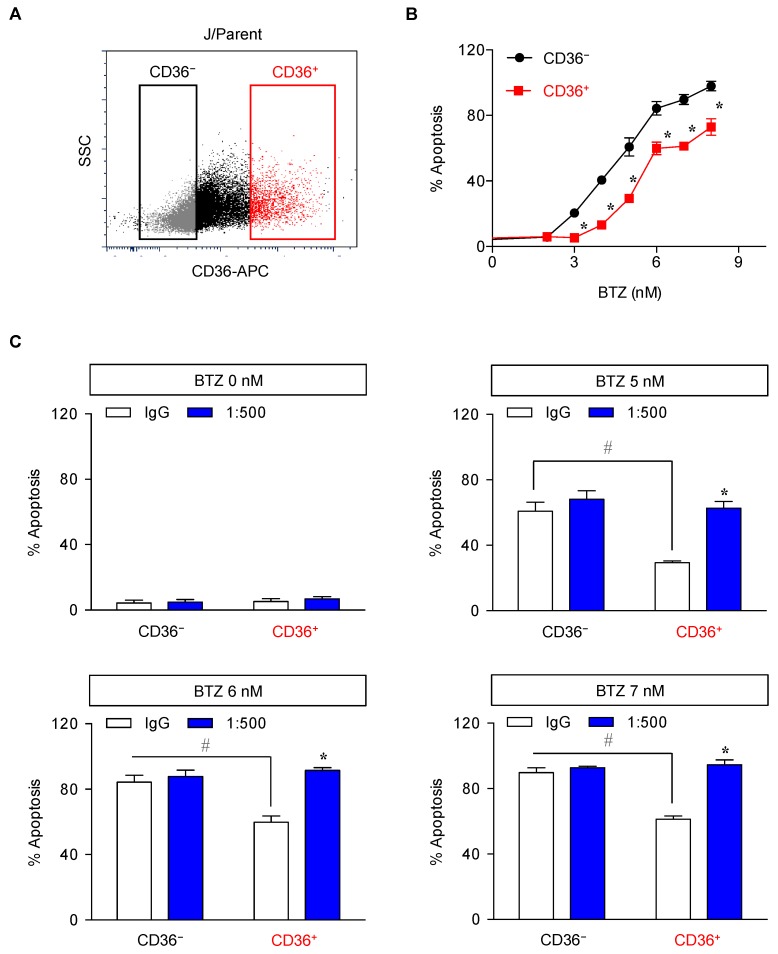
CD36^+^ cells were less susceptible to BTZ-induced apoptosis. Parental (J/Parent) cells were isolated based on CD36 expression into CD36^−^ and CD36^+^ cells using fluorescence-activated cell sorter (FACS) (**A**) and apoptosis in response to BTZ (0–8 nM) were determined by Hoechst 33342 assay at 24 h (**B**). Data are mean ± SD (*n* = 3). * *p* < 0.05 versus CD36^−^ cells; two-sided Student’s *t*-test. (**C**) CD36^+^ cells were pretreated with neutralizing antibody (1:500) for 1 h and treated with BTZ (0–7 nM) for 24 h. After which, apoptosis was determined by Hoechst 33342 assay. Data are mean ± SD (*n* = 3). * *p* < 0.05 versus BTZ-treated IgG control CD36^−^ or CD36^+^ cells; two-sided Student’s *t*-test. ^#^ *p* < 0.05 versus CD36^−^ control; two-sided Student’s *t*-test.

To first evaluate whether CD36 rendered MCL cells to acquire apoptosis resistance to BTZ, we isolated parental J/Parent cells into surface CD36-negative (CD36^−^) and CD36-positive (CD36^+^) cells using fluorescence-activated cell sorter (FACS) (Figure 7A). Figure 7B shows that CD36^+^ cells were less susceptible to apoptosis in response to BTZ (0–8 nM) at 24 h when compared to CD36^−^ cells. To ensure that these differences in BTZ sensitivity were due to CD36 expression, we used anti-CD36 neutralizing antibody (1:500 or 2 μg/mL) to block surface CD36 in CD36^+^ cells. Figure 7C shows that blocking of the surface CD36 increased BTZ-induced apoptosis in CD36^+^ cells to the comparable level to CD36^−^ cells.

**Figure 8:**
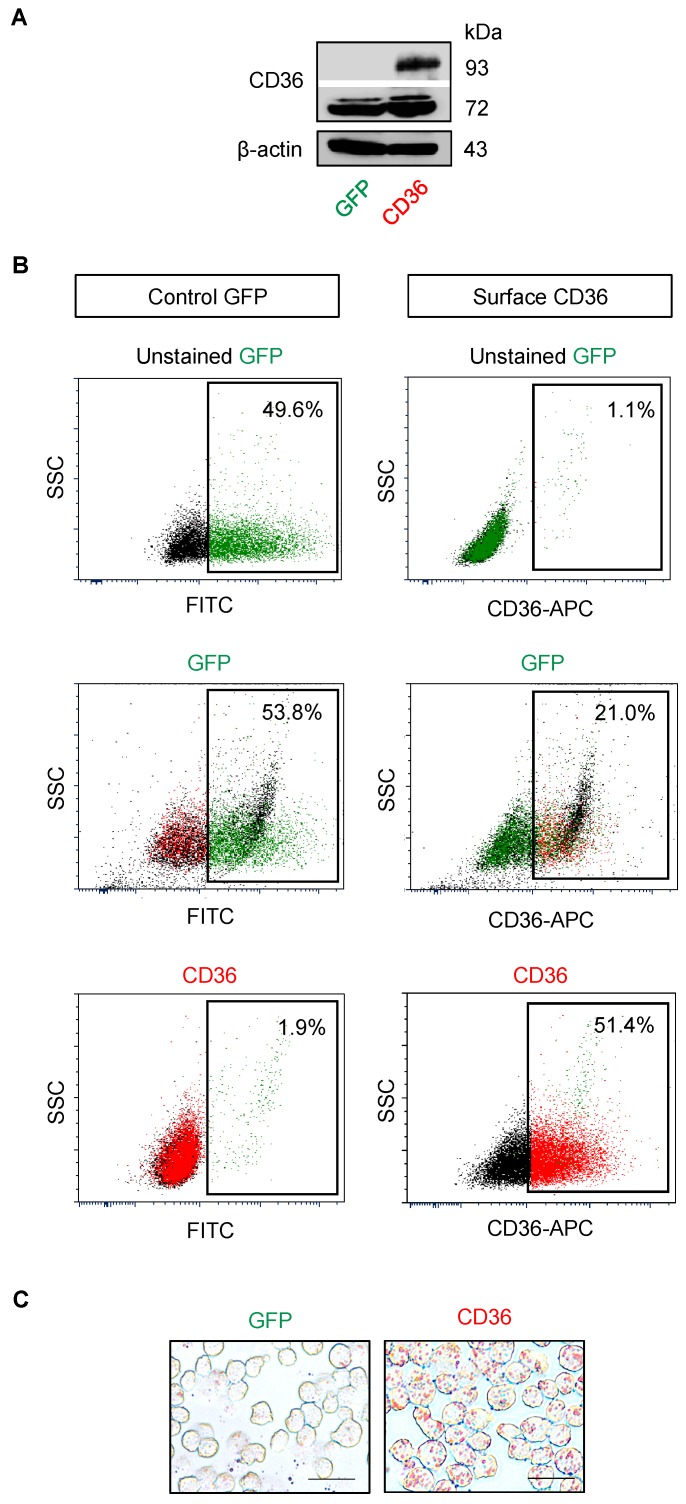
Overexpression of CD36 caused an accumulation of lipid content. Parental (J/Parent) cells were transfected with CD36 or green fluorescence protein (GFP) control plasmid using nucleofection. (**A**) Transfected cells were analyzed for total CD36 level using Western blotting. (**B**) Flow cytometry analysis of surface CD36 (right) and GFP for transfection control (left). Percentage of CD36- and GFP-positive cells (box) were determined based on their internal negative control (unstained cells). (**C**) Analysis of lipid droplets by oil red O staining. Scale bar = 50 µm.

**Figure 9:**
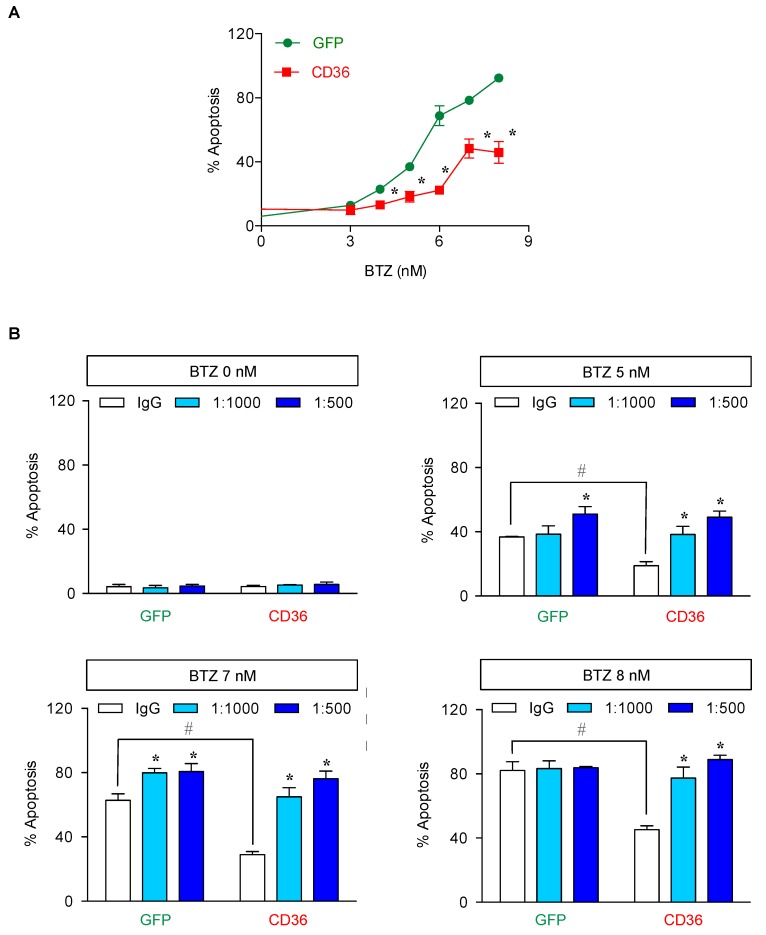
CD36 is a key mediator of BTZ-induced apoptosis. (**A**) Parental (J/Parent) cells were transfected with CD36 or GFP control plasmid using nucleofection and apoptosis in response to BTZ (0–8 nM) was determined by Hoechst 33342 assay at 24 h. Data are mean ± SD (*n* = 4). * *p* < 0.05 versus GFP control; two-sided Student’s *t*-test. (**B**) CD36-overexpressing Jeko-1 cells were pretreated with neutralizing antibody (1:1000–500) for 1 h and treated with BTZ (0–8 nM) for 24 h. After which, apoptosis was determined by Hoechst 33342 assay. Data are mean ± SD (*n* = 3). * *p* < 0.05 versus BTZ-treated IgG control GFP or CD36-overexpressing cells; two-sided Student’s *t*-test. ^#^ *p* < 0.05 versus GFP control; two-sided Student’s *t*-test.

**Figure 10:**
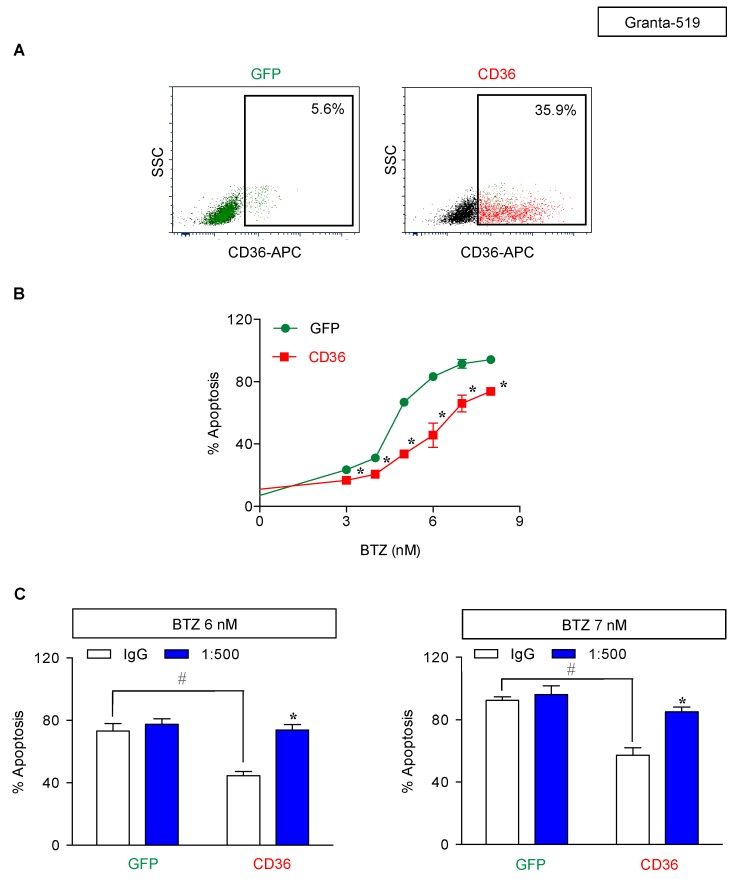
Generality of the role of CD36 in BTZ-induced apoptosis in multiple MCL cell lines. Human MCL-derived Granta-519 cells were transfected with CD36 or GFP control plasmid using nucleofection. (**A**) Flow cytometry analysis of surface CD36. Percentage of CD36-positive cells (box) were determined based on their internal negative control (unstained cells). (**B**) Apoptosis of CD36-overexpressing and GFP control cells in response to BTZ (0–8 nM) were determined by Hoechst 33342 assay at 24 h. Data are mean ± SD (*n* = 3). * *p* < 0.05 versus GFP control; two-sided Student’s *t*-test. (**C**) CD36-overexpressing Granta-519 cells were pretreated with neutralizing antibody (1:500) for 1 h and treated with BTZ (0–7 nM) for 24 h. After which, apoptosis was determined by Hoechst 33342 assay. Data are mean ± SD (*n* = 3). * *p* < 0.05 versus BTZ-treated IgG control GFP or CD36-overexpressing cells; two-sided Student’s *t*-test. ^#^ *p* < 0.05 versus GFP control; two-sided Student’s *t*-test. See also Supplementary Figure S1 for data from human MCL-derived SP49 cells.

Next, we manipulated CD36 in parental cells by ectopic gene expression to ascertain the role of CD36 in BTZ sensitivity. J/Parent cells were transfected with CD36 and its level was determined prior to an evaluation of apoptosis. Western blot and flow cytometric analyses confirmed an increase in total and surface CD36 in CD36-overexpressing Jeko-1 cells (Figure 8A,B). Importantly, an accumulation of lipid droplets, as evaluated by oil red O staining, was apparently observed in CD36-overexpressing cells, while lipid droplets were practically minimum in control transfected green fluorescence protein (GFP) cells (Figure 8C). Figure 9A shows that CD36-overexpressing cells rendered the J/Parent cells to BTZ resistance when compared to GFP cells. To strengthen this finding, GFP and CD36-overexpressing cells were treated with various concentrations of BTZ in the presence or absence of anti-CD36 neutralizing antibody (1:1000–1:500). Anti-CD36 antibody exerted a pronounced sensitizing effect on BTZ-induced apoptosis in CD36-overexpressing cells, and to a lesser extent in GFP cells, in a dose-dependent manner (Figure 9B), thereby validating the role of CD36 in MCL apoptosis protection. Similar findings on the protective role of CD36 were also observed in human MCL-derived Granta-519 and SP49 cells (Figure 10 and Supplementary Figure S1), suggesting the generality of the observed effects in MCL cells.

**Figure 11:**
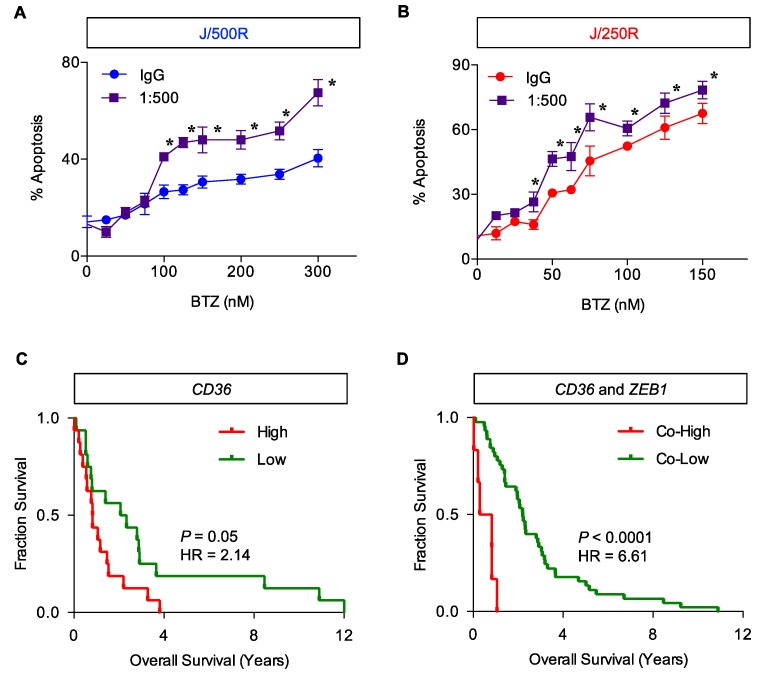
Inhibition of CD36 enhances BTZ sensitivity in BTZ-resistant cells. BTZ-resistant J/500R cells (**A**) or J/250R cells (**B**) were treated with BTZ (0–300 or 0–150 nM) in the presence or absence of CD36 neutralizing antibody (1:500) and apoptosis was determined by Hoechst 33342 assay at 24 h. Data are mean ± SD (*n* = 4). * *p* < 0.05 versus IgG control; two-sided Student’s *t*-test. (**C**) Kaplan Meier survival plot of MCL patients from the gene microarrays (GSE10793) according to the level of *CD36* expression. Overall survival of patients with highest expression (cutoff: Upper quartile, red line) is compared to patients with lowest expression (cutoff: Lower quartile, green line) (*n* = 32). (**D**) Kaplan Meier survival plot of MCL patients (GSE10793) according to the level of *CD36* and *ZEB1* co-expression. Overall survival of patients with highest co-expression (cutoff: Upper quartile, red line) is compared to patients with lower co-expression (green line) (*n* = 54).

To provide supporting evidence on the BTZ sensitization by CD36 inhibition, BTZ-resistant J/500R and J/250R cells were treated with BTZ (0–300 nM) in the presence or absence of anti-CD36 neutralizing antibody (1:500) and apoptosis was determined at 24 h. Figure 11A shows that blocking of CD36 significantly reduced BTZ resistance in J/500R cells. With the basal expression of CD36 in J/250R cells, it is not surprising that the inductive effect of CD36 inhibition on BTZ-induced apoptosis in J/250R cells was also significant, albeit at a lower level than those of J/500R cells (Figure 11B). Altogether, these results strengthen the potential application of CD36 inhibition for BTZ sensitization.

It is well-accepted that drug resistance is dependent on multiple complex mechanisms. Since Zeb-1 has been previously reported by our group to be an important therapeutic target that correlates with patient overall survival and BTZ resistance in MCL via cancer stem cell regulation [32], we next performed database analysis based on mRNA expression of *CD36* and co-expression of *CD36* and *ZEB1* in human MCL microarrays available on Gene Expression Omnibus (GEO; accession number GSE10793) [33] to examine their association to clinical outcome. Our analysis revealed that MCL patients with the highest expression of *CD36* (cutoff: Upper quartile) have shorter overall survival when compared to MCL patients with lowest expression (cutoff: Lower quartile) (Figure 11C). Interestingly, the hazard ratio increased approximately three-fold when compared to the overall survival between MCL patients with highest co-expression of *CD36* and *ZEB1* (cutoff: Upper quartile) to MCL patients with lower co-expression of *CD36* and *ZEB1* (Figure 11D). Notably, the number of MCL patients with lowest co-expression (cutoff: Lower quartile) was not sufficient to perform the analysis. These clinical data support the involvement of CD36, together with Zeb-1, in controlling of aggressive MCL.

## 3. Discussion

MCL is one of the most aggressive lymphomas attributable to its innate and acquired drug resistance that critically limits the clinical outcome in patients [1,2,3,4]. Here, we observed that the development of resistance towards BTZ in MCL was unique, as classical genes involved in drug resistance and lymphoma cell survival were mostly downregulated when compared to their parental cells, opposed of their continued growth (Figure 2A,B). We postulated that this phenomenon might occur due to the global DNA hypermethylation (Figure 2C,D). The association between drug resistance and global hypermethylation has previously been reported [34,35]. In breast carcinoma, chronic intermittent exposure to adriamycin induced hypermethylation that was associated with multidrug resistant phenotype in correlation to an upregulation of *DNMT1*, *DNMT3A* and *DNMT3B* and an increase in DNA methyltransferase activity [36]. DNA demethylation by antisense targeting or hydralazine was capable of restoring breast carcinoma sensitivity to adriamycin and paclitaxel. Similar findings on the role of hypermethylation in the development of drug resistance were observed in neuroblastoma [37,38].

Several studies have considered an aberrant metabolism as a key feature that leads to the emergence of aggressive tumors [39,40]. Here, we used FTIR spectral analysis to generally examine biochemical changes that differentiate between BTZ-resistant cells and parental cells with an intention to find novel candidate targets (Figure 3). We observed the changes in lipid and ester and plausible changes in either nucleic acid, glycoprotein and/or carbohydrate in highly BTZ-resistant J/500R cells that imply the metabolic reprogramming in these cells (Figure 4). The critical linkage between metabolism and BTZ sensitivity has recently been reported by our group via a posttranslational medication in the hexosamine biosynthetic pathway called *O*-GlcNAcylation [41]. An increase of global *O*-GlcNAcylated protein, a form of glycoprotein that is an ideal sensor for nutritional changes, potentiates MCL response to BTZ and reverses the resistant phenotype, consistent with the findings from FTIR analysis.

Dysregulated lipid metabolism has been associated with aggressive forms of numerous solid cancers and served as potential prognostic biomarkers for prostate, breast, ovarian and colorectal cancers [28,29,42]. In the present study, FTIR analysis unveiled for the first time an increase in cellular lipid content in highly BTZ-resistant J/500R cells (Figure 4B), which was validated by oil red O staining (Figure 5A,B). Excessive lipid has earlier been shown to predict cisplatin resistance in bladder cancer [43] and carboplatin resistance in laryngeal carcinoma [44], indicating the involvement of lipid metabolism in drug sensitivity. To further explore the causal relationship between lipid and BTZ resistance at the molecular level, we profiled key players important in the regulation of endogenous and exogenous lipid and identified CD36 as a potential candidate (Figure 5C and Figure 6). CD36, earlier identified as a platelet integral membrane glycoprotein (GPIV) and a receptor for trombospondin-1 (TSP-1), is a member of class B scavenger receptor family. While its intracellular domains are important in localizing CD36 within caveolae and lipid rafts, which accounts for the diversity of signal transduction, its extracellular domains bind to a vast variety of ligands, including lipid ligands such as native and oxidized lipoproteins, anionic phospholipids and fatty acids [45,46]. Hence, CD36 has subsequent impact on lipid metabolism. We found that overexpression of CD36 caused an increase in cellular lipid droplets (Figure 8), indicating that CD36 promotes lipid uptake and/or accumulation in MCL cells.

Correlated with invasion of tumors and metastasis, CD36 has been extensively proposed as a prognostic biomarker for various types of cancers, mostly of epithelial origin [45,47]. However, the precise roles of CD36 in hematologic malignancies, particularly the regulation of apoptosis and drug response, are still very limited. In the present study, we provided direct evidence that CD36 protected various human MCL-derived cells against BTZ-induced apoptosis (Figure 7, Figure 9, Figure 10 and Supplementary Figure S1). Blocking of surface CD36 using neutralizing antibody strongly reversed the protective effect of CD36 in parental cells and importantly reversed the resistant phenotype in BTZ-resistant cells (Figure 11A,B), highlighting the potential application of CD36 inhibition for BTZ sensitization. CD36 antibody therapy has recently been shown to reduce cancer severity in preclinical models of human orthotopic oral cancer and prostate cancer patient-derived xenografts, supporting the clinical significance of antibody-based therapeutic targeting of CD36 [47,48]. Our findings are substantiated by survival analyses of MCL patients showing poor clinical outcomes with high *CD36* expression that becomes even worse with the co-expression of *ZEB1* (Figure 11C,D). It is worth noting that *CD36* expression was, however, negatively associated with overall survival of multiple myeloma patients [49].

Zeb-1 (also known as TCF8) is a known zinc finger transcription factor and an EMT activator. In MCL, it has been shown to promote tumor growth in vivo and correlated with poorer clinical prognosis [20]. We earlier found that Zeb-1 prevents various MCL cells against BTZ-induced apoptosis through cancer stem cell expansion and maintenance [32]. Since Zeb-1 has been identified as a driver of adipogenesis in 3T3-L1 fat cell differentiation [50], we evaluated the association between Zeb-1, CD36 and lipid. Overexpression of Zeb-1, although induced lipid accumulation in MCL cells, had minimal effect on CD36, and vice versa (Supplementary Figure S2). These results suggest that Zeb-1 and CD36 independently mediate lipid reprogramming and BTZ response in MCL.

## 4. Materials and Methods

### 4.1. Cell Culture and Reagents

Human MCL-derived Jeko-1 cells were obtained from American Type Culture Collection (ATCC; Manassas, MA, USA), while Granta-519 and SP49 cells were kind gifts of Dr. Siwanon Jirawatnotai (Systems Pharmacology, Faculty of Medicine Siriraj Hospital, Bangkok, Thailand) [51,52]. Mycoplasma contamination was checked every eight weeks using MycoAlert mycoplasma detection kit (Lonze, Cologne, Germany) and any cell lines found positive were discarded. Cells were cultured in RPMI1640 medium containing 10% fetal bovine serum (FBS), supplemented with 2 mM L-glutamine, 100 U/mL penicillin and 100 μg/mL streptomycin, and maintained in a humidified atmosphere of 5% CO_2_ environment at 37 °C. BTZ was obtained from Janssen-Cilag (Beerse, Belgium) and a stock solution of 1 mg/mL was produced and stored at −20 °C until use. Hoechst 33342 was obtained from Molecular Probes (Eugene, OR, USA). Antibody for CD36 for flow cytometry was obtained from BioLegend (San Diego, CA, USA), while antibodies for neutralization and Western blotting were from Abcam (Cambridge, UK). All other antibodies were obtained from Cell Signaling Technology (Beverly, MA, USA) and all other reagents, including DAC and oil red O, were obtained from Sigma-Aldrich (St. Louis, MO, USA).

### 4.2. Generation of De Novo BTZ-Resistant Cells

BTZ-resistant cell lines were generated by stepwise selection method as described previously with slight modifications [13]. Parental MCL-derived Jeko-1 (J/Parent) cells were continuously exposed to increasing concentrations of BTZ to the maximum concentration of 100, 250 and 500 nM. Resistant cells were analyzed by Annexin V-FITC/PI assay, where double negative cells were sorted using BD FACSAria cell sorter (BD Biosciences, San Jose, CA, USA) and designated as J/BTZ100R, J/BTZ250R and J/BTZ500R, respectively. Dead Cell Removal Kit (Miltenyi Biotec, Auburn, CA, USA) was used to remove the dead cells during culture. The dose-response curve of BTZ on resistant cells at different degrees was plotted and LC50 was calculated using GraphPad Prism software (La Jolla, CA, USA) as previously described [53].

### 4.3. Apoptosis Assay

Apoptosis was determined by Hoechst 33342 assay. Briefly, cells were incubated with 10 μg/mL Hoechst 33342 for 30 min and analyzed for apoptosis by scoring of cells having condensed (brighter than non-apoptotic) and/or fragmented nuclei by fluorescent microscopy (Eclipse Ti-U with NiS-Elements, Nikon, Tokyo, Japan). The apoptotic index was calculated as the percentage of cells with apoptotic nuclei over total number of cells.

### 4.4. RNA Isolation and Quantitative PCR

Total RNA was prepared using TRIzol reagent (Invitrogen, Carlsbad, CA, USA). Isolated RNA was reverse-transcripted with High-Capacity cDNA Reverse Transcription kit (Applied Biosystems, Foster City, CA, USA). Quantitative PCR analysis was carried out on CFX384 Real-Time PCR (Bio Rad, Hercules, CA, USA) using a Power SYBR Green PCR master mix (Applied Biosystems). Initial enzyme activation was performed at 95 °C for 10 min, followed by 40 cycles of denaturation at 95 °C for 15 s and primer annealing/extension at 60 °C for 1 min. Relative expression of each gene was normalized against the housekeeping *GAPDH* gene product. Heatmap and cluster analysis were performed using MultiExperiment Viewer (MeV) software (http://mev.tm4.org/).

### 4.5. FTIR Sample Preparation and Microspectroscopy Analysis

A drop of 5 μL of 4 × 10^5^ cells was deposited onto IR-transparent 2-mm-thick barium fluoride windows, air dried, and stored in a desiccator until spectra were acquired. The spectra were acquired at BL4.1 IR Spectroscopy and Imaging Beamline at the Synchrotron Light Research Institute with a Vertex 70 FTIR Spectrometer (Bruker Optics, Ettlingen, Germany) coupled with an IR microscope (Hyperion 2000, Bruker Optics) and a mercury-cadmium-telluride (MCT) detector cooled with liquid nitrogen over the measurement range from 4000 to 800 cm^−1^. The microscope was connected to a software-controlled microscope stage and placed in a specially designed box that was purged by dry air. The measurements were performed using an aperture size of 10 × 10 μm with a spectral resolution of 4 cm^−1^, with 64 scans co-added. Spectral acquisition and instrument control was performed using OPUS 7.2 software (Bruker Optics). Spectra from each sample group were analyzed by using PCA. Data were preprocessed by performing a baseline correction and then normalized using Extended Multiplicative Signal Correction using the spectral regions from 3000–2800 cm^−1^ and 1800–900 cm^−1^ using Unscrambler 10.1 software (CAMO, Oslo, Norway). Next, unsupervised hierarchical cluster analysis of FTIR spectra data sets of the regions from 3000–2800 cm^−1^ to 1750–800 cm^−1^ was performed using Ward’s algorithm, which utilized a matrix defining the inter-spectral distances to identify the two most similar FTIR spectra. The spectral distances between all of the remaining spectra and the new clusters were then recalculated using OPUS 7.2 software.

### 4.6. DNA Methylation Status

Global DNA methylation was determined using the MethylFlash^TM^ Methylated DNA Quantification Kit (Epigentek, Farmingdale, NY, USA), according to the manufacturer’s protocol. In brief, 100 ng genomic DNA was prepared and bound to assay wells that were specifically treated to have high DNA affinity. After which, methylated DNA (5-mC) was captured by specific antibody and signal was enhanced and quantified colorimetrically by measuring an absorbance at 450 nm using a microplate reader (Synergy H1, BioTek, Winooski, VT, USA). Relative 5-mC level was expressed as the ratio of signals from the resistant and control samples.

### 4.7. Oil Red O Staining and Quantification

Cell suspension at 2 × 10^5^ cells were collected onto a 24-well plate, washed twice with phosphate buffered saline (PBS) and fixed with formalin vapor for 15 min. After fixation, the cells were washed twice with distilled water, air dried and stained with filtered oil red O working solution (60% oil red O stock solution in distilled water; stock solution, 5 mg/mL oil red O powder in isopropanol) for 20 min at room temperature. The cells were then washed twice with distilled water to remove an unbound dye and visualized under a microscope. After microscopic examination, the amount of oil red O was quantified in each well by extracting the dye using isopropanol and gently pipetting and its absorbance was read at 510 nm using a microplate reader (Synergy H1, BioTek).

### 4.8. CD36 Staining and Flow Cytometric Analysis

For surface staining, cells at the density of 2 × 10^5^ cells/100 μL in PBS were labeled with 2 μL of APC-conjugated antibody against CD36 (BioLegend, San Diego, CA, USA) for 15 min at room temperature. The cells were then washed, fixed in 2% paraformaldehyde for 30 min and resuspended in PBS for analysis by flow cytometry. For intracellular staining, cells were first fixed in 2% paraformaldehyde and permeabilized with 0.5% Tween-20 in PBS for 15 min, followed by antibody incubation.

### 4.9. Plasmids and Transfection

mCherry-CD36-C10 (CD36) plasmid was a gift from Prof. Michael Davidson (Addgene # 55011). Cells were transfected with CD36 or GFP control plasmid by nucleofection using 4D-Nucleofector^TM^ (Lonza, Morristown, NJ, USA) with EW113 (Jeko-1 cells) or with DN100 (Granta-519 and SP49 cells) device program. The transfected cells were analyzed for CD36 prior to use by flow cytometry and/or Western blotting.

### 4.10. Western Blotting

After specific treatments, cells were incubated in a commercial lysis buffer (Cell Signaling Technology) and a protease inhibitor mixture (Roche Molecular Biochemicals, Indianapolis, IN, USA) at 4 °C for 30 min. Protein content was analyzed using bicinchoninic acid (BCA) protein assay (Pierce Biotechnology, Rockford, IL, USA) and 50 μg of proteins were resolved under denaturing conditions by sodium dodecyl sulfate polyacrylamide gel electrophoresis (SDS-PAGE) and transferred onto polyvinylidene difluoride (PVDF) membranes. Membranes were blocked with 5% nonfat dry milk, incubated with appropriate primary antibodies at 4 °C overnight, and subsequently incubated with peroxidase-conjugated secondary antibodies for 1 h at room temperature. The immune complexes were analyzed by enhanced chemiluminescence detection system on a digital imager (ImageQuant LAS, GE Healthcare, Pittsburgh, PA, USA).

### 4.11. Gene Microarray Dataset and Survival Analysis

The published dataset GEO ID GSE10793, which were obtained from tumor biopsies from 71 untreated MCL patients, was used to calculate overall survival associated to Zeb-1 and CD36. Genechip measurements were performed using NCI/Staudt human 15K v13 arrays [33]. Kaplan Meir survival plot of MCL patients was generated using GraphPad Prism software according to the level of gene expression with the cutoff value for the highest expression at upper quartile.

### 4.12. Statistical Analysis

The data represent means ± SD from three or more independent experiments as indicated. Statistical analysis was performed by two-sided Student’s *t*-test at a significance level of *p* < 0.05.

## 5. Conclusions

In conclusion, we demonstrate here the involvement of lipid metabolism in the regulation of apoptosis in MCL cells. Increased lipid content by means of lipid droplets, in part via the upregulation of CD36, reduced BTZ sensitivity of MCL cells, causing BTZ resistance. Therefore, lipid accumulation and CD36 overexpression could be important in predicting therapeutic responses of MCL. We further advise the potential application of CD36 inhibition in combination therapy to battle against drug-resistant MCL. Additionally, we also suggest the use of FTIR spectroscopy as a promising technique in cancer research, particularly when exploring the molecular mechanisms involved in metabolic reprogramming and drug resistance.

## Supplementary Materials

The following are available online at https://www.mdpi.com/2072-6694/11/4/576/s1. Figure S1: CD36 is a key mediator of BTZ-induced apoptosis, Figure S2: Overexpression of Zeb-1 and CD36 in MCL Jeko-1 cells, Table S1: FTIR Band Assignment for Biological Samples.
