# Supplementary material for: Deciphering the Elevated Lipid via CD36 in Mantle Cell Lymphoma with Bortezomib Resistance Using Synchrotron-Based Fourier Transform Infrared Spectroscopy of Single Cells
Source: Cancers (Basel). 2019 Apr 24;11(4):576. doi: 10.3390/cancers11040576 (PMC6521097; doi:10.3390/cancers11040576)
Supplement: Supplementary file 1 [file cancers-11-00576-s001.pdf]

Supplementary Materials

Deciphering the Elevated Lipid via CD36 in Mantle Cell Lymphoma with Bortezomib Resistance Using Synchrotron-Based Fourier Transform Infrared Spectroscopy of Single Cells

Sudjit Luanpitpong, Montira Janan, Kanjana Thumanu, Jirarat Poohadsuan, Napachai Rodboon, Phatchanat Klaihmon and Surapol Issaragrisil

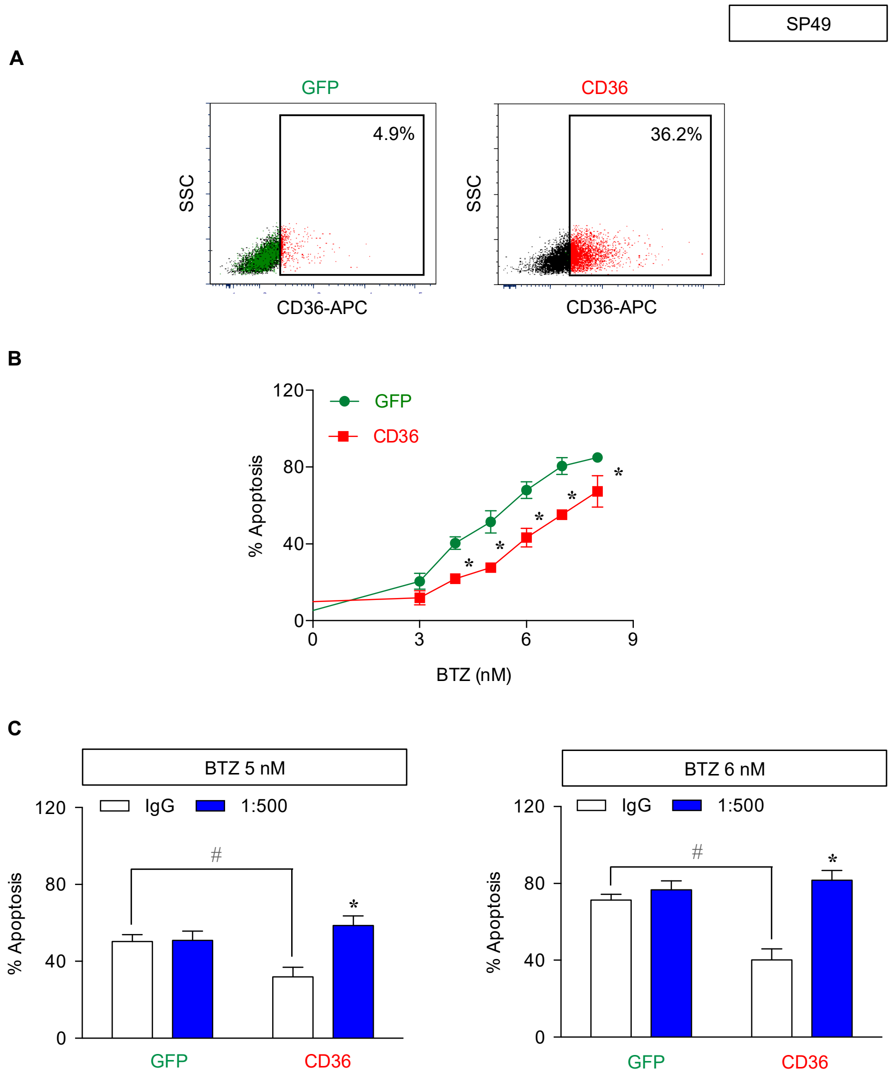

Figure S1. CD36 is a key mediator of BTZ-induced apoptosis. Human MCL-derived SP49 cells were transfected with CD36 or GFP control plasmid using nucleofection. (**A**) Flow cytometry analysis of surface CD36. Percentage of CD36-positive cells (box) were determined based on their internal negative control (unstained cells). (**B**) Apoptosis of CD36-overexpressing and GFP control cells in response to BTZ (0–8 nM) were determined by Hoechst 33342 assay at 24 h. Data are mean ± SD (n = 3). * $p < 0.05$ versus GFP control; two-sided Student's t-test. (**C**) CD36-overexpressing SP49 cells were pretreated with neutralizing antibody (1:500) for 1 h and treated with BTZ (0–6 nM) for 24 h. After which, apoptosis was determined by Hoechst 33342 assay. Data are mean ± SD (n = 3). * $p < 0.05$ versus BTZ-treated IgG control GFP or CD36-overexpressing cells; two-sided Student's t-test. # $p < 0.05$ versus GFP control; two-sided Student's t-test.

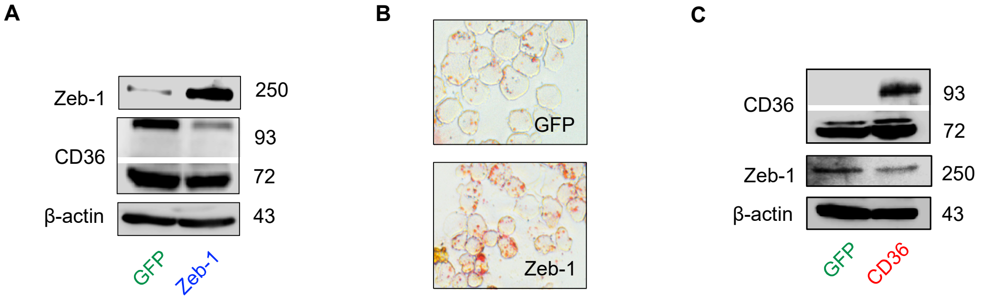

Figure S2. Overexpression of Zeb-1 and CD36 in MCL Jeko-1 cells. (**A**) Parental Jeko-1 cells were transfected with Zeb-1 or GFP control plasmid using nucleofection and levels of Zeb-1 and CD36 were evaluated by Western blotting. (**B**) Analysis of lipid droplets by oil red O staining in Zeb-1-overexpressing cells. (**C**) Jeko-1 cells were similarly transfected with CD36 or GFP control plasmid using nucleofection and levels of CD36 and Zeb-1 were evaluated by Western blotting.

Table S1. FTIR Band Assignment for Biological Samples.

Wavenumber (cm^{-1})	Assignment
3000–2800	C–H stretching mainly from lipid
1750–1735	C=O ester mainly from lipid
~1700–1600	Mainly γ(C=O) associated with protein as the amide I band
1685	Beta turn protein secondary structure
1652	α-helix protein secondary structure
1635	β-sheet protein secondary structure
~1600–1500	Amide II (protein N–H bend, C–N stretch), polysaccharide, glycoproteins
~1250,1080	P=O phosphodiester group from nucleic acid
~1160,1060,1037	C–O vibrations from glycogen and other carbohydrates, glycogen and other carbohydrate
~1121,954	C–C vibrations from RNA ribose chain nucleic acid

© 2019 by the authors. Licensee MDPI, Basel, Switzerland. This article is an open access article distributed under the terms and conditions of the Creative Commons Attribution (CC BY) license (http://creativecommons.org/licenses/by/4.0/).
